# Seeing It from Both Sides: Do Approaches to Involving Patients in Improving Their Safety Risk Damaging the Trust between Patients and Healthcare Professionals? An Interview Study 

**DOI:** 10.1371/journal.pone.0080759

**Published:** 2013-11-06

**Authors:** Susan Hrisos, Richard Thomson

**Affiliations:** Institute of Health & Society, Newcastle University, Newcastle upon Tyne, United Kingdom; University of St Andrews, United Kingdom

## Abstract

**Objective:**

Encouraging patients to be more vigilant about their care challenges the traditional dynamics of patient-healthcare professional interactions. This study aimed to explore, from the perspectives of both patients and frontline healthcare staff, the potential consequences of patient-mediated intervention as a way of pushing safety improvement through the involvement of patients.

**Design:**

Qualitative study, using purposive sampling and semi-structured interviews with patients, their relatives and healthcare professionals. Emergent themes were identified using grounded theory, with data coded using NVIVO 8.

**Participants:**

16 patients, 4 relatives, (mean age (sd) 60 years (15); 12 female, 8 male) and 39 healthcare professionals, (9 pharmacists, 11 doctors, 12 nurses, 7 health care assistants).

**Setting:**

Participants were sampled from general medical and surgical wards, taking acute and elective admissions, in two hospitals in north east England.

**Results:**

Positive consequences were identified but some actions encouraged by current patient-mediated approaches elicited feelings of suspicion and mistrust. For example, patients felt speaking up might appear rude or disrespectful, were concerned about upsetting staff and worried that their care might be compromised. Staff, whilst apparently welcoming patient questions, appeared uncertain about patients’ motives for questioning and believed that patients who asked many questions and/or who wrote things down were preparing to complain. Behavioural implications were identified that could serve to exacerbate patient safety problems (e.g. staff avoiding contact with inquisitive patients or relatives; patients avoiding contact with unreceptive staff).

**Conclusions:**

Approaches that aim to push improvement in patient safety through the involvement of patients could engender mistrust and create negative tensions in the patient-provider relationship. A more collaborative approach, that encourages patients and healthcare staff to work together, is needed. Future initiatives should aim to shift the current focus away from “checking up” on individual healthcare professionals to one that engages both parties in the common goal of enhancing safety.

## Introduction

Many experts in the field believe that patients have a role in making their own care safer [1-5]. Evidence suggests that many patients are not only willing and able to take a role in improving their safety [6,7], but also that such a role could improve safety [8-13]. A number of current initiatives exist that promote patient involvement in improving their own safety, either by encouraging broad vigilance across all aspects of their healthcare (e.g. “Speak Up” (USA) [14]; “Please Ask” (UK) [15]; or by targeting specific safety issues (e.g. infection control ) [16,17]. A common goal of these patient-mediated approaches is to “push” improvement by promoting patient identification and reporting of safety concerns. However, concerns have been raised about the potential of such approaches to impact negatively on public trust in healthcare [18-20], patient trust in their providers, the patient-provider relationship and on staff morale [21-26]. 

Recent empirical work exploring patients’ preference and willingness to engage in their healthcare and the uptake of safety behaviours, suggests that willingness can vary depending on the nature and severity of their illness [8,27], the type of safety issue or recommended action [28,29], the type of question – “factual” or “challenging” [23,28-31], their sense of capability or self-confidence [7,26], and the type of healthcare professional the patient is interacting with [30,32-34]. The few qualitative studies exploring the patient perspective provide crucial insight into the reasons behind patients’ and relatives’ hesitance and reported comfort levels associated with engaging in both safety behaviours and in other aspects of their healthcare, such as shared decision making. Fears of being rebuffed and of potential retribution, and the need for provider permission, are consistently elicited barriers to engagement [32,35-39]. 

A small but growing number of studies have aimed to understand the healthcare professionals’ perspective in relation to patient involvement in improving safety [40-45]. This recent literature provides very valuable and important insight into healthcare professionals’ attitudes towards patient involvement in the prevention of error [40,42-44], and interventions aiming to promote such a patient role [41,45]. This work suggests that healthcare professionals, like patients, generally view patient involvement positively. However, it also suggests that professionals’ approval or support can vary depending on the nature of the behaviour being promoted [41]. For example, though doctors felt it to be important that patients ask healthcare staff about hand washing, they were less willing to support this patient behaviour. In another study, oncology nurses who again viewed patient involvement in improving the safe administration of their chemotherapy drugs favourably, described engaging patients in this endeavour as “challenging” [42]. Nurses in the latter study also expressed feelings of frustration when error prevention involving patients had failed. In a more recent study, the same authors found that whilst healthcare professionals’ approved of patient participation in two error prevention behaviours (asking staff if they had washed their hands and identifying medication error) they viewed the effect this may have on patient-professional relationship more negatively [40]. These findings provide an important first insight into the professional perspective regarding potential unwanted or negative consequences of promoting a patient role in improving their own safety. However, there remains little in-depth understanding of such concerns, particularly in relation to healthcare professionals, and there are no qualitative studies to date that explore these issues from both the patient and professional perspective. The aim of the current analysis was therefore to address this gap in the literature by exploring the perceived advantages and disadvantages of promoting a patient role in improving patient safety, from the perspectives of both service users (patients and their relatives) and frontline healthcare staff. 

## Materials and Methods

### Ethical statement

This study was approved by the UK NHS National Research Ethics Service Newcastle and North Tyneside 2 Research Ethics Committee (REC reference number 10/H0907/24. Study title: “Promoting patient involvement in improving safety: A qualitative development study”) in May 2010. 

The analysis presented here is based on data collected as part of a wider qualitative study, conducted to inform the development of an intervention to promote patient and relative involvement in improving their own safety. A semi-structured topic guide [Appendix S1], primarily focussed on exploring novel ways in which service users might play a role in improving their own safety, was used to elicit respondent views about current approaches to promoting patient involvement in improving safety. The topic guide explored respondents’ understanding of “patient safety”; beliefs and attitudes towards patient involvement in improving their safety; what respondents felt was a feasible and acceptable role for patients; and how such a role might be best supported. Example materials from a range of current campaigns (UK and international) aiming to encourage patients to take a more active role in improving their safety were presented to respondents during the interview. A summary of example patient behaviours promoted by these campaigns is provided in Appendix S2. Purposive sampling was employed to capture a broad range of perspectives across different healthcare contexts. Eligible respondents were a) patients who had recent experience as an inpatient on one of eight participating wards within two urban NHS Foundation Trust hospitals in North East England; b) their relatives or carers and c) staff providing care to patients on these wards (doctors, nurses, health care assistants and pharmacy staff). Wards were purposively selected to provide access to a range of both acute and elective medical and surgical patients. Patients who did not speak English and patients who lacked capacity were excluded. Relatives of patients lacking capacity were eligible to take part. Patient and relative participants were initially identified prior to discharge by a research nurse or patient representative, who introduced the study to them using the study information leaflet. Contact details of patients expressing an interest were provided to SH who then telephoned patients one week post-discharge to complete recruitment. Interviews were conducted in respondents’ own homes. Staff participants were initially identified by ward leads, guided by the purposive sampling frame. Potential staff participants were provided with an information leaflet and asked to contact the study researcher if they were interested in taking part. The venue for staff interviews was at the choice of the interviewee, but all interviews took place on hospital premises. SH undertook all interviews and signed consent was obtained from each participant at the outset of their interview. Staff interviews, took, on average, 30 minutes, and patient interviews one hour. All interviews were audio-recorded and transcribed verbatim. 

Interview transcripts were analysed iteratively by SH using a grounded theory approach [46]. supported by the use of NVIVO [47]. Emergent themes were discussed in depth with RT to develop a coding frame and to guide avenues of exploration in subsequent interviews. Codes were added or revised as new themes emerged [48,49]. Preliminary findings were further discussed during regular project team meetings that included two lay patient and public involvement members, as well as clinical representatives from each of the two participating hospitals. Interviews continued until data saturation was reached, i.e. when no new themes were emerging. The resonance of key themes was checked during group sessions with patients, relatives and ward staff, some of whom had previously participated in an interview. 

## Results

### Sample Characteristics

16 patients (10 female, six male) and four relatives (two female, two male) took part, ranging in age from 25 to 79 years (median 62 years). Eleven of these respondents had left full-time education before the age of 18, the remaining had completed either work-based professional training (n=4) or (as a minimum) an undergraduate degree (n=5). Five patients had an existing chronic illness; with three being admitted due to exacerbation of this. Six patients were emergency admissions (three medical and three surgical), and 10 were elective admissions (a mix of general and orthopaedic planned surgery). Three elective patients had received treatment for a new, life-threatening condition. Three relatives were the spouse of the patient, and one was the parent. Relatives reported on three acute patients, one elective. Interviews were undertaken jointly for two patient/relative dyads. All patient and relative respondents described themselves as “White, British”. Thirty-nine healthcare staff also took part in an interview (9 pharmacists, 11 doctors, 12 nurses and 7 health care assistants). Eighteen doctors, nurses and HCAs were based on surgical wards and 12 on medical wards, pharmacy staff worked across wards. Length of time qualified ranged from 2 years to 42 years (median 11years). Each healthcare professional group interviewed included senior (i.e. consultants, modern matrons, senior pharmacists), middle-grade (i.e. registrars, nursing sisters, ward pharmacists) and junior (i.e. F2 doctors, staff nurses, pharmacy technicians) staff respondents. Two members of staff were of other European origin and three were of non-European origin.

### Emergent Themes

#### Perceived advantages of patient involvement in improving their own safety (Table 1)

**Table 1:** Perceived advantages of patient involvement in improving patient safety.

**Patients & Relatives**
“Understanding what is happening”“
“They [patients] should know what to expect. Having information is a great empowerer isn’t it? If you know what should be happening to you then you can have some influence at the point of something taking place.” (Patient1 M Aged 76)
“You are wanting to understand why certain things are being done… reassurance about why they’re taking certain actions…” (Patient2 F Aged 78)
“It makes such a difference … [we are] happy for it to be done but [we] want to know why. And some of them [staff] just got on, did it and you’re thinking, ‘Well why was that done? Why was that necessary?’ it’s fear of the unknown. If the patient was given more information.... well, in my son’s case and mine, we would be a lot happier.” (Relative1 F Aged 69)
***“Knowingwhattoexpect”***
“It is just little things like that [who to speak to, how to order meals] which I think … would help you feel as if you fitted in ... you know what to expect and you know what is expected of you as well.” (Patient3 M Aged 67)
“I didn’t understand everything, so I’ve asked the questions to find out what’s going on… what’s going to happen. They stopped my fluids but didn’t tell me… I was wandering around with an empty bag for about 12 hours. if, when they put that bag up, they’d have said, ‘this is your last bag’, I’d have known...” (Patient4 F Aged 26)
**Staff**
***Job efficiency***
“I think information is the first thing to get across…because we have got the advantage [when] the patient knows ‘I’m going in and I am having that done’ - you can prepare them for what’s happening, what to expect … so that they can be actively involved… can help … and I think with their involvement it’s a much easier way.” (Staff Nurse, F, Staff16)
“it is an opportunity for us to tell them about their medicines, to get some sort of concordant agreement that they understand the medicines sufficiently to want to adhere to them… they go home more empowered … if the patient is really well informed they can pick up queries or flag up if they think there has been a mistake.” (Pharmacist, F, Staff21)
“The first thing will be empowerment. I think most doctors and nurses ought to respond positively to someone saying ‘actually I’m worried about this’ you know, ‘rightly or wrongly I am worried about this, can we discuss it?’. And certainly I think one of the things that holds up good communication from the medical point of view is people saying ‘I want to check this’, people initiating it. There’s often the assumption made that because someone’s not asking questions they don’t have any questions or concerns. (Surgical Registrar, M, Staff14)
***Improved compliance***
“I think empowering patients to feel free to challenge us, and ask those questions, is definitely good. Personally speaking when patients ask questions it is fantastic because it shows they are interested. They are more likely to listen to your answers than if I just stand there and tell them what I need to tell them…if they ask a question it’s certainly a two way thing. I believe that they are more likely to retain whatever information I give them.” (Consultant, M, Staff1)
“In my experience, it [involving patients] often makes them more compliant with what you’re trying to achieve, em, because if they feel as though they’re involved, you know, …it makes people more keen to reach a common goal…rather than ‘I’m having things done to me’, you know.” (Senior Nurse, F, Staff6)

Study participants from all groups were generally positive towards the notion of patient involvement in improving their safety and identified potential advantages. For example, patients and their relatives said that they welcomed the opportunity to be able to ask questions or have their concerns addressed, since this provided them with reassurance and a better understanding of what was happening to them and what to expect. As well as helping them to feel “more part of things”, understanding what was happening and knowing what to expect was also seen by patients as essential to them taking a role in improving their own safety. Staff said that they welcomed questions from patients since “it shows that they are interested in their care”. Perceived advantages expressed by staff were improved adherence to treatment and greater patient satisfaction with care, achieved through better understanding. A further advantage of encouraging patients, especially “the quiet ones”, to engage with staff whilst they are in hospital was of being able to “nip problems in the bud”. A common scenario for wards was of patients waiting until they were discharged before reporting concerns, by which time issues that could have been resolved (and harm mitigated) had escalated. Despite this general positivity, subsequent discussion of potential ways in which patients and their relatives might make a contribution to improving their own safety, revealed that some currently recommended behaviours or actions were problematic. 

#### Concerns about involving patients in improving their own safety (Table 2)

**Table 2:** Concerns about patient involvement in improving patient safety.

**Patients & relatives**
***Actions involve challenging or criticising staff***
“I think anything that I have to say I would take as an implied criticism of their professional judgement, so the woman who handed me the tablets, you know, that she’d been sloppy … [and], the guy who took my observations, I guess I would have felt I was questioning his judgement” (Patient5 M Aged 31)
“There were short comings in delivery of care and I kind of stood back initially because I didn’t want to come across as, you know, the consultant kind of ‘know all’. It might have been worse because you’re medical, because they’re, they’re seeing you as being judgemental of them and, I have been in that position and no one likes to be, to be judged as not doing things correctly.” (Staff15 M Aged 45, speaking as a relative)
“No I wouldn’t do it. I expect hospitals to have certain standards and cleanliness is but one of those standards and for me to be asking “have you washed your hands?” or whatever I think it is an insult to the professionalism of the people involved “ (Patient3 M Aged 67)
‘Erm...well it think it's quite – I wouldn't find it easy to do at all. I don't know why but I just – I don't know, I just found the erm nurses so helpful and nice and everything that I don't know, you just didn't like to criticise them in anyway, you know’ (Patient7 F Aged 70)
**Staff**:
***Stafffeelchallenged,criticised***,
“The other negative is sometimes staff feel a bit threatened because if they don’t know what they’re being asked, or they don’t know where to go for help, that’s when you have to have your wits about you and you have to know your subject” (Senior Nurse, F, Staff3)
“I think expert patients still generally terrify us a little bit; partly because it’s so difficult to get accurate information from the internet for example.” (Pharmacist Staff2)
“I have had people [staff] come up to me and say ‘Oh I can’t stand that man’ you know ‘if I tell him once more what I’m doing, I’m the nurse here!’ and I mean we all have those kind of days - me included….” (Senior Nurse, F, Staff3)
“It’s really hard because you’ve got such a mixture of … nice caring people … who work in the health care profession [then] you’ve got others who just don’t accept being told about their job er you know?” (Healthcare Assistant, F, Staff37)
***Staff feel scrutinised, demoralised***
“I think it would sort of be helpful [to involve patients] but I do think a lot of staff would think ‘well they are checking up on my work’ … there would be a really big barrier put up between healthcare professionals and the public really … the majority of the public have just got no trust in the NHS whatsoever.” (Healthcare Assistant, F, Staff45)
“I think you feel that you are being criticised really because you think people are looking for problems and they are looking for complaints. Because I think nurses a lot of the time do get a lot of bashing in hospitals, you know what I mean? (Senior Nurse, F, Staff43)
“The problem is that em … you do have some families who will nit-pick on absolutely everything and the more that that is encouraged the greater the nit-picking that goes on … well that sort of thing does em, nit-picking damage, hugely damages staff morale.” (Consultant, M, Staff15)
“It wasn’t something I expected when I came into the NHS – how overbearing it feels sometimes [feeling of being scrutinised] … and people [staff] write more when they feel like they’re being scrutinised. Which is a shame … we talk about building trust, building partnerships and things like that but it is a little bit difficult.” (F2 doctor, M, Staff39)

In particular, patients and relatives expressed concerns about actions that encourage them to “check” that their care is being delivered correctly and appropriately, and engaging directly with healthcare professionals if they think something is not quite right. Within this context of pushing improvement through patient-mediated intervention, pointing out potential errors or oversights in care provision was felt to be “questioning” or challenging the professionalism of healthcare staff. The discomfort expressed by patients was primarily related to their perceptions of these behaviours as being critical or judgemental of staff, or that staff would interpret such user intervention as criticism. Several staff identified with this patient perspective, sometimes drawing on personal experience of being a patient or the relative of a patient. Asking healthcare professionals if they had washed their hands was particularly problematic, with most patients stating that this was something they probably or definitely would not do. Of those patients who said they would not have a problem in asking this question, doing so was contingent upon other factors – for example that their intervention could be justified by a strong concern (sometimes based on previous experience of hospital acquired infection) or observation that hand washing had not occurred. Other actions perceived by patients and relatives as “challenging” or as “criticising” included overtly or explicitly checking that the correct medicines had been administered during drug rounds and asking about alternative treatment options to those recommended by their doctor. 

For frontline healthcare staff, demands on their time and increased workload burden were very prominent concerns. However, their accounts also mirrored the patient perception that active patient identification of safety concerns involves “checking up on” and “challenging” healthcare staff. This concern was expressed by some staff as anxiety about being asked questions to which they might not know the answers and by others as a frustration at having their professional status or integrity challenged. Staff also spoke of feeling overly scrutinised by both service-users and the system within which they work and how this negatively impacts on staff morale. 

In aiming to understand respondents’ beliefs underlying their concerns, we asked them to elaborate on their misgivings and what they felt might be the consequences of patients doing things they perceived to be confrontational. Several related themes were identified but one key common theme emerged from the accounts of patients, relatives and healthcare professionals, namely risk of damage to the patient provider relationship.

#### Risk of damage to the patient-provider relationship (Table 3)

**Table 3:** Risk of damage to the patient-provider relationship.

**Erosion of patient trust in the competency of the healthcare professional**
**Patients & Relatives: “Life in their hands”; “Doing the job properly”**
“When I go into hospital, literally, you put your life in their hands. That’s what I do, it is called total trust, I trust them. They know their job, they are professionals, if they make a mistake and it hasn’t harmed me then I just don’t know about [it] do I?” (Patient6 M Aged 66)
“I don’t think I would ask [*if HCP had washed hands*]”… we shouldn’t have to ask them – it’s their job …” (Patient11 F Age 60)
“I would not be expecting to do that [*check medications*]… I go in [to hospital] on the basis of relying on the professionalism of the people there.” (Patient14 M Aged 77)
**Staff: “Losing trust / confidence in care providers”; “losing trust / confidence in services”**
“[Patients] will be aware of mistakes that can be made and will lose their trust in the nurses and doctors. I would definitely make sure everything’s in place and - be more wary that they don’t fully trust my skills and my judgement if they are constantly aware of the risks” (Staff Nurse, F, Staff27)
“Patients can get scared by things, if you’re pointing out these things may happen to them they’re going to automatically think that they [will]. I think that’s the negative part of it, because people pick up on it and think it happens everywhere” (Senior Nurse F Staff12)
“It is kind of finding a balance ‘cause I think if you scare them too much are they going to want to come into hospital? or are they going to, you know, try and get out of hospital quicker than they maybe should?” (Pharmacist, F, Staff36)
**Erosion of healthcare professional trust in the “good” patient.**
**Patients & Relatives: “Patient will be labelled difficult”; “Overstepping traditional boundaries"**
“They’re [staff] always rushing about doing, like 101 jobs on their shift bless them, because they are hard worked; sometimes it’s not wanting to bother them. I didn’t [ask] at first because I didn’t want to look [like] I was being pushy” (Patient8, F, Aged 40)
“Oh … ‘who does she think she is - I’m the person in charge here’... ‘she doesn’t know anything about medical issues’. I’m probably entirely wrong, that’s the way I feel as an ordinary lay person, that you should know what you’re talking about before you start querying it” (Relative1, F, Aged 69)
“You don't want to get a reputation for yourself as a difficult patient ... the nursing staff are going to go away and talk about you at the main desk, sort of ‘oh god, he's on about this again’. I think my fear, not so much with the nursing staff, more the doctors themselves, was this idea of this sort of ‘armchair doctor’, I haven’t got a medical degree; all I've got is an enquiring mind and access to the internet. And my worry is that, here you are faced with people with years of medical training and you're saying, on the basis of a Wikipedia entry, ‘well actually I think this drug might be better for the following reasons’. It's sort of well ‘who am I to say that?’”. (Patient5, M, Aged 31)
**Staff: “Patient will be labelled difficult”; “Suspicion of patient motives”**
“Well they always get labelled er ‘awkward patients’ kind of thing. You know ‘he’s always asking this, he’s always asking that’ then they sort of get a stigma attached to them in the end.” (Staff Nurse, F, Staff27)
“Now and again you get someone who will get a book out and they are writing something down... it’s the first thing that you think… especially if it's a relative … if somebody is more concentrated on writing down the time and what the name is and da de da when the relative is ill - that’s looking for something you know…”. (Senior Nurse, F, Staff43)
“I think a lot of staff are on the defensive yeah, definitely yeah … everybody is frightened that they are going to get a complaint because that’s all you hear now... ” (Healthcare Assistant, F, Staff45)

Erosion of patient trust in the competency of the healthcare professional: Patients often commented that they “had to put their trust” in those providing their care, because “you put your life in their hands” when you go into hospital. Whilst there is an element of “hope” within this, there was also a general expectation that healthcare providers “know what they are supposed to be doing” and a common assumption that they always did what they were supposed to do. For example, washing their hands appropriately, knowing that the leg they were about to operate on is the correct one, or giving out the correct medications were deemed part of the professional’s ‘job’. Some patients felt (quite strongly) that they “shouldn’t have to ask” about such things, expressing a fundamental reliance on healthcare providers to deliver care professionally. Whilst such perceptions are clearly linked to role expectations, this stance resonates with popular conceptualisations of trust within the context of healthcare, wherein (patient) trust in their care provider(s) is associated with expectations of goodwill and competence on the part of those trusted [21]. This suggests that some patients may experience a loss of trust in the competency or integrity of their care providers, if they feel that they “have to” ask or tell them about potential lapses in their care, because they are not doing the job properly. This concern was mirrored by some healthcare professionals who felt that raising awareness about safety issues might erode patient trust in them as competent practitioners and reduce confidence in the services they provide. 

Erosion of healthcare professional trust in the “good” patient: Patients and relatives expressed concern that they, in the context of identifying safety concerns, might be perceived less favourably by care providers by appearing rude, offensive or critical, especially when the patient or relative had (on the face of it) a good rapport with their care provider. In addition to the risk of damaging a good rapport with individual staff, this concern was also linked more broadly to the risk of being labelled as “difficult”. Patients and relatives were generally hesitant about asking “too many questions” as they did not want to be seen as “pushy” or “awkward”. This sense of insecurity and mistrust in care providers was also apparent in the belief that “demanding” patients might be “discussed by staff” and thus flagged to other colleagues on the ward. Being labelled in this way was something that healthcare staff confirmed as a potential risk for enquiring service-users. Linked to patients’ reluctance to offend was a similar reluctance to overstep what might be understood as traditional role boundaries. Checking or asking if providers were “doing their job correctly” or appearing to contradict their clinical judgement, was felt by some to be “insulting their professionalism” and that it was “not the place” of the “lay” service-user. These beliefs were expressed even if the patient or relative felt that they were the better informed party, and were also evident in the accounts of healthcare professionals speaking from their experience as patients and relatives. Quite notably there was a common tendency amongst staff to talk about patients and relatives asking questions in the context of complaining rather than information seeking or sharing. Some staff further reported being suspicious of patients and relatives who asked questions, raised concerns or wrote things down, and questioned their motives; interpreting such actions as preparation for making or backing up a complaint. This was despite a general understanding and empathy amongst staff for the service-users’ need for information. 

A seemingly very important concern for patients was that healthcare professionals would somehow “treat the patient differently” as a consequence of upsetting them and the balance of rapport between them. Further probing revealed that this might happen in two ways: fear of being rebuffed or chastised; and fear that care might be somehow compromised.

#### Staff may treat the patient “differently” (Table 4)

**Table 4:** Staff will treat the patient differently.

**Fear of being rebuffed or chastised**
**Patients & Relatives: “HCPs might not respond favourably”; “assessing staff receptiveness”; “feeling vulnerable”; “staff behaviours”**
“II don’t think I would say anything to be honest I would be frightened to offend them in case they got upset and you would be thinking well they could be a bit awkward with you .. . you can tell the different ways nurses act and doctors, the way they are with you... we had nurses on there who were absolutely amazing and you would have a good laugh with them … and then you would get some that would be like ‘move, we’ve got to get that done,’ and you would be like “God it’s like you are in the army”. They were not very approachable - with them you would be like ‘I’m not saying a word because she has a chance to bite my head off’. (Patient9, F, Aged 52)
“I think it differs probably more according to personality than according to rank or function … I've had consultants that I've been very happy to ask questions of because the tone they set early on is one of sort of acceptance and erm engagement, and then I've had other consultants who you kind of feel are edging to get away and just get you done and dusted as soon as possible…I've still asked [questions] but I've not felt comfortable doing it.” (Patient5 M Aged 31)
“On a different day, … I would have dealt with it [*Consultant’s attitude*], but because it was about me I didn’t feel like I could …, I guess part of that might be confidence that you’re right and they’re wrong, because… there is something about being a patient that makes you feel tiny and inconsequential and inadequate …” (Patient4 F Aged 26)
“What you want to do when you, when you get onto a ward, even like a visitor, you want to get on well with the staff. Go in there and talk to them and em, and … speak to them in a ‘hey, you know, you haven’t done this’ - not in a nasty way.” [Relative2 M Aged 58]
**Staff: *HCPsactively&passivelychastising***
“Sometimes…they [relatives] can maybe just ask something and sometimes one of the staff will turn around and be quite funny back and you think ‘well’ ” (Healthcare Assistant, F, Staff45)
“I think sometimes nurses do, if a patient rings the buzzer for something that we feel is insignificant say, some trivial issue [*trivial in our eyes*]… I think by the way we respond to that em often outside, well sometimes in our body manner and tone of voice when we talk to them we indicate that it is not popular “ (Staff Nurse, M, Staff41)
**Fear of care being compromised**
**Patients & Relatives: “HCPs might not look after patient well”; *HCPs will be less empathetic***
“I basically don’t want to upset this nurse because she’s looking after [patient] … and if I say something to upset her she might not look after [patient] as well as she would have done if I hadn’t said something”. (Relative2 M. Aged 58)
“In case, you know, their attitude towards me changed … because I think it does happen.” [Then, in relation to an incident kept from spouse] “[Spouse] wouldn’t [be concerned about asking] but I was the one that was lying in the bed, you know…” (Patient15 F Aged 58)
“I mean not that they would be [but] your treatment could be different somehow, you know ‘I’ll just leave her a bit, she’s cheeky her’… tell the other nurses ‘watch her - fussy woman over there’. Do you understand what I mean? That type of thing”. (Patient9 F Age 52)
**Staff: *HCPs speaking as patients & relatives***
“You’ll hear patients when there’s no doctors around going ‘I can’t believe he did this’, ‘that was inappropriate’, they’ll sit in the waiting room and they’ll shout the odds about why [the clinic] is running behind or whatever … they know it’s wrong, they know something needs to be done about it, but they don’t want it to affect their treatment, and I totally understand that, you know I think we’re all like that to some extent…”. (Patient providing dual perspective as a healthcare professional)
“My [relative] was in hospital - and bear in mind that I am a senior person in the organisation and I have worked in health - but there were questions I was un-keen to ask…. Em worst thing I suppose to say - although I can’t imagine this being the case - but if you ask, if you are a difficult patient or you are a difficult relative will that somehow compromise my care? I’m not suggesting anything would be done like that but em it is that sort of thought you know, ‘If I am seen as being a trouble making family’ em you know ‘does it mean that you actually get a better level of care or a worse level of care?’ It is a terrible thing to think but it is basically what goes through your head.” (Healthcare professional reflecting experience as relative)

Fear of being rebuffed or chastised: Healthcare providers were expected to always remain “professional” in their dealings with patients and their families, regardless of the situation, and there appeared to be a general consensus amongst both patients and healthcare professionals that most would. However, there was a common belief that, some care providers might not respond favourably to being asked a seemingly challenging or critical question. Being rebuffed or chastised was a very real fear for many patients, and a key barrier to them speaking up. In assessing the potential receptiveness of staff, patients described being acutely sensitive to the perceived “demeanour” and personality of those providing their care and how staff interacted with them, their relatives, other patients, and other staff. The uncharacteristic, sometimes extreme, vulnerability that patients appear to experience whilst in hospital serves to intensify this heightened sensitivity. It was apparent from staff accounts however, that chastising patients and relatives does happen, and that this may be done both actively and passively. 

Fear that care might be compromised: An additional belief expressed by patients was that some staff “may not look after” the patient as well as they had done before if patients or their relatives upset them. Respondents struggled to articulate exactly *how* they felt that a patient’s care might be compromised and were eager to clarify that they didn’t really believe that staff would do anything so blatantly inappropriate as to cause them harm. Patients expected that they would still receive adequate “basic care” on the basis of an (accountable) professional duty, or code of conduct. It was felt that healthcare staff might become less attentive and perhaps even (deliberately) make patients wait for aspects of care – for example by being slow to respond to a bell call or by making patients wait for assistance in bathing. More generally though the concern again focussed on relationship issues - in that staff (regardless of whether they overtly demonstrated offense or not) might be less empathetic or sincere in their interactions with patients or less likely to act on or take their concerns seriously. It is notable that these concerns were shared by healthcare professionals when reflecting on their own experience of being a patient, or the relative of a patient.

#### Behavioural implications of service-user fears (Table 5)

**Table 5:** Behavioural implications.

**Patients**
“I think the only way I’d feel comfortable doing that [ask if HCP had washed their hands] was if I actually caught a bad infection whilst in hospital, and I think then it would encourage me to say.” (Patient8 F Aged 40)
“There was one night nurse that I was apprehensive about … I hadn’t got any medication … she did get me morphine but … she wasn’t a very nice person, I didn’t like her she was very em, brusque - looked as if she hated the job … I didn’t want to ask her anything that was going to make her any more cross than she already was (laughs)” (Patient2 F Aged 78)
I would [ask] because it’s my meds and I don’t want them putting something into my body that’s not right, but I would probably, you know if they put me on a drip and I didn’t know what it was for, I would wait until the nice one came in and say, ‘what’s this?’, em so you’d save any question that could wait, for the nice ones.” (Patient16 F Aged 70)
**Staff**
“I noticed it more when I went into a Sister’s uniform, I was documenting everything, I was making my own little statements. Every conversation I was having with a relative - because they do, things get twisted, things are not heard right… you are encouraged [to keep notes] but you can go a bit ridiculous I don’t do that now…” (Senior Nurse, F, Staff43)
“Sometimes … I think it is just the news and things like that - everybody sort of has their heckles up don’t they … everybody is frightened that they are going to get a complaint because that’s all you hear now it’s just. You do watch everything that you are doing because you think they will complain ...” (Healthcare Assistant, F, Staff45)
“My longest documentation is when I discuss stuff with relatives. Because I write down what I’ve said, what they said; I quote stuff. I mean, it’s a bit tricky, especially in the kind of society we live in these days given that it’s so litigious… We’re doing that to defend ourselves if a patient turns around and sues. Um… so I think it would be very hard for health care services to kind of view a patient’s keeping detailed records as anything but that, that’s why we do it. That’s an absolute baseline (F2 Doctor, M, Staff39)
“Some [staff] like not to give very much information ‘cos it means they don’t get very many questions and people haven’t got to think of answers … [and] some nurses – especially newly qualified nurses – think they’ve got to do everything, you know; and that’s when you panic and mistakes happen” (Staff Nurse, Staff3)

For some patients, the perceived consequences of upsetting staff, and disrupting relationships, were so powerful that they admitted not sharing potentially serious queries or concerns even with their relatives, who they knew would immediately raise them with staff (Table 4; Patient 15). Others suggested that it would take contracting an infection to make them feel sufficiently empowered (and justified) to ask a healthcare provider to wash their hands. Patients suspecting that a member of staff might be unreceptive to being asked questions, or for assistance, reported avoiding interactions with these individuals. Arguably such avoidance, in a similar way to not sharing concerns with their relatives, serves to put patients at greater risk of harm. Furthermore, this “self-protection strategy” was not restricted to those who feared being left feeling emotionally “bruised” by being rebuffed or who feared retribution. For some it was simply an understandable preference to interact with “nice” or more pleasant staff. An additional consequence of this, that warrants mention, is the potential increased burden on “nice” staff, since patients will purposely wait to target them with their queries. 

#### Behavioural implications of healthcare professional fears (Table 5)

Some staff suggested that they and their colleagues could become guarded in their interactions with certain patients and their relatives, therefore distancing themselves from being the potential target of a complaint. Amidst such concerns, provider accounts also demonstrated evidence of the adoption of other self-protective behaviours. For example, in addition to “distancing” themselves, some staff reported: keeping detailed records of interactions and conversations with patients or relatives suspected to be planning a complaint; of limiting opportunities for patients and/or relatives to ask questions; and of providing only limited information.

## Discussion

Current understanding of patients’ reluctance to engage proactively in aspects of their healthcare and healthcare safety is largely based on the experience of disease specific populations and/or specific patient safety behaviours [19,50-52]. The work presented here provides new and deeper insight into what beliefs underlie patients’ general concerns about asking questions or alerting staff to possible oversights in their care. Novel understanding is also provided, of how patients and relatives perceive that their involvement might impact negatively on their experience of care and their interactions with healthcare staff. To our knowledge, this is the first qualitative enquiry that compares and contrasts patient and provider perspectives in the same analysis. Our findings are also of topical importance in light of a recent independent inquiry into the poor care provided by a UK NHS hospital Trust [53], which found service users’ were reluctant to insist on having their concerns addressed, even in the face of serious failings, for fear of “upsetting staff”. A further unique and important contribution of the current work is the identification of potential behavioural implications that may actually increase patients’ risk of harm whilst in hospital. Both patients and staff described actively avoiding prolonged interactions with each other: patients with unreceptive staff, and staff with enquiring patients. Evidence is also presented to suggest that patients are prepared to withhold important concerns, even from relatives, to protect themselves from potential retribution. However, our data also reveal a second, but equally important, aspect to concerns expressed in relation to “upsetting staff”, in that the perceived consequences of this are not limited to being overtly rebuffed. Concern about upsetting or offending “nice”, receptive staff, and subsequently disrupting an established positive rapport, can potentially work in the same way. These findings provide some empirical support for the conclusions of a recent systematic literature review, whose authors suggest that patients may assume a passive role in their healthcare as a means of actively protecting their personal safety [54], It is of further note that reticence to engage directly with healthcare staff is apparent regardless of educational level and/or professional level occupation. This finding was echoed recently in a qualitative study of patient participation in shared decision making in the USA [55]. 

We also observed that healthcare professionals, when speaking from experience as patients or relatives, express the same misgivings, regardless of their seniority. This somewhat contradicts recent quantitative work, which suggests that healthcare professionals may be more willing to participate (as patients) in safety related behaviours [43]. The latter study, as many previous studies, also found that staff viewed patient involvement positively. Staff in our study did too, but as suggested by our more in-depth analysis, this positivity may be in more general terms and its reporting could be influenced by socially desirable responding. Other recently published quantitative work does suggest that healthcare professionals too anticipate a negative effect on the patient-provider relationship as a result of patient involvement in improving safety [40]. This is an important finding that lends support to those reported here, but quite what the anticipated effect might be or why healthcare professionals should feel this way cannot be inferred from survey data. Many current patient-mediated approaches encourage patients to engage directly with healthcare staff if they have concerns or questions about their care. This intrinsically requires the patient, or relative, to interact with and speak to staff; to either ask them something or to tell them something. Our findings reveal both practical and emotive reasons why patients are reluctant to directly engage with staff in this way and why staff may view such interactions negatively. Our data further support and expand findings emerging from quantitative work (e.g. 40) that both patient and staff attitudes towards patient safety behaviours can vary depending on the nature of the behaviour and that this in turn will influence their willingness to engage in them. Asking staff if they have washed their hands is a particular example of a less favourable safety behaviour that can be perceived as “challenging” or confrontational, whilst patients asking staff questions about their care and what to expect was perceived more favourably by the patients and staff in the present study. This resonates with studies that have shown the acceptability of questioning behaviours to be more favourable for questions that are factual in nature [23,28-31]. 

In the present analysis, perceptions of being challenged and scrutinised were apparent in the accounts of staff. This may lend some explanation for the anticipation of a negative effect on the patient-provider relationship suggested by the healthcare staff who took part in Schwappach et al’s vignette based study [40]. These authors found that staff approval not only varied differentially for two “challenging” behaviours (asking about hand washing was seen less positively than the identification of medication error), but that positive staff attitudes towards the behaviours were mainly determined by the manner in which patients intervene and whether the patient intervention correctly identified an error. In addition, the present work uniquely illuminates a vulnerability experienced by staff, by uncovering salient beliefs that appear to undermine healthcare professionals’ desire and motivation to embrace and support patient involvement. Staff concerns about patients losing confidence in them, maintaining professional integrity, and their misplaced distrust in some patient actions, warrants careful consideration if efforts to promote patient involvement in improving patient safety are to succeed. This is particularly relevant given the emphasis on the importance of the relational aspects of care delivery apparent in our respondent groups and that the need for provider permission is a consistently elicited barrier to patient engagement in general [32,35,36,39,55,56].

### Implications for practice

Patient-mediated approaches to improving quality and safety that prompt patients to “challenge” healthcare professionals by asking them questions or by “speaking up” when they have concerns, rarely consider the needs of healthcare staff. Not preparing staff for “activated” and “empowered” patients runs the risk of damaging staff morale and of negatively impacting on professional practice. A trusting patient-provider relationship is considered to be the cornerstone of successful healthcare delivery [57-64]. We have shown that elements of approaches that push improvement through the involvement of patients inadvertently threaten to undermine this fundamental foundation by creating reciprocal suspicion and doubt between patient and provider. This questions the suitability of such “one-sided” approaches that can appear to “pitch” patients against healthcare professionals, eroding trust in the competency of healthcare staff and in the services they deliver. Potentially avoidable harm may occur even when individual healthcare professionals are highly motivated, knowledgeable and skilful in their delivery of healthcare [21]. Entwistle & Quick (2006) propose a conceptual framework for a new understanding of trust in healthcare relationships that acknowledges that healthcare professionals are human and make mistakes [21]. The framework dispels the healthcare provider as “perfectionist” myth and encourages openness whilst discouraging blame. The authors argue that patients can be both vigilant and trusting partners in their care & safety. 

### Implications for research

Patients who are more comfortable about engagement appear to be more likely to take action in error prevention behaviours [28,32]. Our findings raise concerns about damage to staff morale and a perceived threat to professional integrity. Taken together, this clearly indicates that future approaches to promoting patient involvement in improving patient safety should seek to engender confidence and mutual trust in both patients and care providers. Research should therefore aim to design and implement interventions that promote a collaborative approach by supporting the needs of both parties. This position is supported by a small but growing call for more focus on the relational and subjective factors that enable patient participation [21,54,65-68]. We propose that a new emphasis on “de-sensitising” patient safety behaviours, commonly perceived as “challenging” or “checking up on” healthcare professionals, is needed, and believe this is essential to the development of a valuable patient role that is seen as routinely necessary and complementary to achieving a common aim. Partnering with patients and families to improve safety through such culture change is further justified and supported by the findings of the recently published public inquiry into the failings of the UK Mid-Staffordshire NHS Trust [69] and the subsequent responses to this report by the UK Government [70], and the UK Health Foundation [71], that emphasize the need for significant cultural change that puts the patient first and foremost in care delivery. 

### Strengths & Weaknesses

A particular strength of this study is that patient, relative and healthcare staff perspectives, focusing on the same phenomenon, are compared and contrasted. This approach has provided a strong contextualisation of existing knowledge, as well as enabling new insight into important dynamics that may influence patient and provider tolerance of safety improvement efforts. Whilst not generalisable beyond the sample studied, the findings resonate with the available literature. Furthermore, sampling of a generic range of patients and healthcare staff, from a variety of both acute and elective specialties provides a rich, grounded account of experience that makes a unique and important contribution to current understanding. 

## Conclusions

Healthcare professionals, patients and relatives generally perceived separate and mutually positive benefits of involving patients in improving their safety, but they also shared important fears and anxieties. Amidst a sense of suspicion and mistrust, both patient and professional accounts suggested the adoption of potentially counter-productive behaviours that could exacerbate patient safety problems. These reveal the critical need for a collaborative, mutually acceptable, approach to patient involvement in the promotion of safety improvement, and a more contemporary conceptualisation of “trust” between patients and providers. Active support and intervention needs to occur simultaneously for patients, relatives and healthcare professionals to prevent misunderstanding and unwanted consequences. 

## Supporting Information

Appendix S1 **Patient & Staff Interview Topic Guides.**
(DOC)

Appendix S2 **Examples of patient safety behaviours promoted by current campaigns.**
(DOCX)
