# Supplementary material for: Seeing It from Both Sides: Do Approaches to Involving Patients in Improving Their Safety Risk Damaging the Trust between Patients and Healthcare Professionals? An Interview Study
Source: PLoS One. 2013 Nov 6;8(11):e80759. doi: 10.1371/journal.pone.0080759 (PMC3819291; doi:10.1371/journal.pone.0080759)
Supplement: Appendix S1 — Patient & Staff Interview Topic Guides. (DOC) [file pone.0080759.s001.doc]

# **Title of Project: Promoting patient involvement in improving safety**

**Patient Interview Topic Guide**

1. **Respondent’s understanding of ‘patient safety’**

*Prompts*

*What do you understand by the term ‘patient safety’ – what do you think I mean/I am talking about when I use that term?*

*Clarify*

1. **Respondent 's experience of being a patient within the NHS and their beliefs and attitudes about safety**

*Prompts*

*First time in hospital?*

*Any concerns* *about going into hospital? If yes – why, what concerns?*

*Did anything to try to minimise own risk?*

*Did anything happen whilst in hospital that made you feel unsafe or worried? If yes - Did anything about it? If yes - What? If did nothing - Why not?*

1. **Thoughts about the idea that patients have a role to play in enhancing their safety whilst in hospital**

*Prompts*

*How do you feel about the idea that patients can contribute to their safety?*

*Probe experience of current initiatives/campaigns*

*Should patients help keep themselves safe when in hospital?*

**4. Ways in which respondent thinks patients [or their relatives or carers] could contribute to enhancing their safety** [spontaneous ideas]

*Prompts*

*What can patients [carers, relatives] do to help keep themselves safe?*

*How do you think hospital staff might feel about the efforts you describe/suggest?*

*What sorts of things do you think might stop patients/put them off/encourage them doing any of the things you suggest?*

*How confident or comfortable would you feel about doing any of these things?*

**5. Ways in which respondent thinks patients can or cannot contribute to enhancing their safety** [recommended approaches]

*Present list of examples of currently suggested/recommended patient behaviours & some example materials*

*Prompts*

*How comfortable/confident would you be about doing any of these recommendations?*

*What are advantages/disadvantages in asking patients to do these things?*

*How might these recommended behaviours/actions change things?*

*How supportive might staff be towards patients to do these recommended behaviours?*

*How might we encourage patients to do any of these things?*

**6. Anything else you would like to add?**

# **Title of Project: Promoting patient involvement in improving safety**

**Ward Staff Interview Topic Guide**

**1. Respondent’s understanding of ‘patient safety’**

*Prompts*

*What do you understand by the term ‘patient safety’ – what do you think I mean/I am talking about when I use that term?*

*Clarify*

**2. Respondent 's beliefs and attitudes about patient safety**

*Prompts*

*What do you think are the risks that patients face when they go into hospital?*

*Are they preventable?*

*What causes them?*

*What kinds of things do hospitals and Drs and nurses do to keep patients safe?*

**3. Thoughts about the idea that patients have a role to play in enhancing their safety whilst in hospital**

*Prompts*

*How do you feel about the idea that patients can contribute to their safety?*

*Probe experience of current initiatives/campaigns*

*Should patients help keep themselves safe when in hospital?*

**4. Ways in which respondent thinks patients [or their relatives or carers] could contribute to enhancing their safety** [spontaneous ideas]

*Prompts*

*What sorts of things can patients [carers, relatives] do to help keep themselves safe?*

*How do you think patients might feel about the efforts you describe/suggest?*

*What sorts of things do you think might stop patients/put them off/encourage them to do any of the things you suggest?*

*How confident or comfortable would patients feel about doing any of these things?*

**5. Ways in which respondent thinks patients can or cannot contribute to enhancing their safety** [recommended approaches]

*Present list of examples of currently suggested/recommended patient behaviours & some example materials (campaign leaflets, patient guidelines)*

*Prompts*

*What are advantages/disadvantages in asking patients to do these things?*

*How might these recommended behaviours/actions change things?*

*How supportive would you [staff] be of these recommended behaviours?*

*What needs to done to encourage patients to do any of these things?*

*How best might this be done [to achieve a mutually acceptable, collaborative approach to improving safety]?*

*How comfortable/confident would you be about patients [carers, relatives] doing any of these recommendations?*

**6. Anything else you would like to add?**
