# Supplementary material for: Seeing It from Both Sides: Do Approaches to Involving Patients in Improving Their Safety Risk Damaging the Trust between Patients and Healthcare Professionals? An Interview Study
Source: PLoS One. 2013 Nov 6;8(11):e80759. doi: 10.1371/journal.pone.0080759 (PMC3819291; doi:10.1371/journal.pone.0080759)
Supplement: Appendix S2 — Examples of patient safety behaviours promoted by current campaigns. (DOCX) [file pone.0080759.s002.docx]

**Appendix 2: Examples of patient behaviours promoted by current patient safety campaigns**

**Minimise risk of harm arising from own actions**

• Helping to reach an accurate diagnosis

• Sharing decisions about treatments & procedures

• Knowing their medications & why they take them

• Taking medicines as prescribed

• Reporting side effects or other changes in condition

• Participating in infection control initiatives

**Minimise risk of harm arising from others’ actions**

- Paying attention to the care they are being given
- Observing & checking procedures
- Ask the doctor to mark the site of an operation
- Ask caregivers if they have washed their hands
- Speaking up if they have any questions or concerns
- Telling a caregiver if they think there has been a mistake in their treatment or medication
